# A Comparison of Homogenization vs. Enzymatic Lysis for Microbiome Profiling in Clinical Endoscopic Biopsy Tissue Samples

**DOI:** 10.3389/fmicb.2018.03246

**Published:** 2019-01-08

**Authors:** Chao Zhang, Prashant V. Thakkar, Sarah Ellen Powell, Prateek Sharma, Sreekar Vennelaganti, Doron Betel, Manish A. Shah

**Affiliations:** ^1^Institute for Computational Biomedicine, Weill Cornell Medicine, New York, NY, United States; ^2^Division of Hematology and Medical Oncology, Department of Medicine, Weill Cornell Medicine, New York, NY, United States; ^3^Veterans Affairs Medical Center and University of Kansas School of Medicine, Kansas City, MO, United States

**Keywords:** gastrointestinal tract, whole genome sequencing, quantitative PCR, microbiome, clinical biopsy, metagenomics, DNA extraction

## Abstract

Identification of the human microbiome has proven to be of utmost importance with the emerging role of bacteria in various physiological and pathological processes. High throughput sequencing strategies have evolved to assess the composition of the microbiome. To identify possible bias that may exist in the processing of tissue for whole genome sequencing (WGS), it is important to evaluate the extraction method on the overall microbial content and composition. Here we compare two different methods of extraction, homogenization vs. enzymatic lysis, on gastric, esophageal and colorectal biopsies and survey the microbial content and composition using WGS and quantitative PCR (qPCR). We examined total bacterial content using universal 16S rDNA qPCR as well as the abundance of three phyla (*Actinobacter, Firmicutes, Bacteroidetes)* and one genus (*Fusobacterium)*. We found minimal differences between the two extraction methods in the overall community structure. Furthermore, based on our qPCR analysis, neither method demonstrated preferential extraction of any particular clade of bacteria, nor significantly altered the detection of Gram-positive or Gram-negative organisms. However, although the overall microbial composition remained very similar and the most prevalent bacteria could be detected effectively using either method, the precise community structure and microbial abundances between the two methods were different, primarily due to variations in detection of low abundance genus. We also demonstrate that the homogenization extraction method provides higher microbial DNA content and higher read counts from human tissue biopsy samples of the gastrointestinal tract.

## Introduction

The human microbiota is an integral part of human physiology, influencing human development, host immunity, and nutrition (Shreiner et al., 2015). Despite extensive studies conducted on the human microbiome over the past decade, the precise ecological relationship between microbial constituents and their human hosts is not fully understood. An important step in the evaluation of the human microbiome is to accurately extract and quantify microbial content from human tissues (Rossmanith and Wagner, 2011). The human microbiome is remarkably diverse, not only among different individuals but also between anatomic sites within a single host (Human Microbiome Project, 2012).

Differences in extraction methodology may lead to differences in microbial identification due to differences in cell wall structures and susceptibility of certain microbial species to various lysis strategies. Different enzymes lead to variable cell lysis efficiencies for different species (Yuan et al., 2012). Peptide cross-links in peptidoglycan layers within cell walls determine the extent to which some bacteria are more or less resistant to lysing methods vs. mechanical ones (Moore et al., 2004; Lazarevic et al., 2013). As such, there is no single “gold standard” by which to determine microbial composition, especially for low-abundance human biopsy samples from differing anatomic sites.

Most of the upper gastrointestinal (GI) tract, especially the stomach, is a notoriously depauperate environment in terms of microbial composition and diversity, as compared to the oral cavity and the lower gastrointestinal tract. The stomach is a particularly unique and challenging locale from which to isolate and examine microbial constituents because of increased acidity and mucus production, which generally discourages microbial colonization. A number of methods are described in the literature with varied results in diversity, species selection and abundance (Wu et al., 2010; Yuan et al., 2012; Crandall et al., 2016). Few studies to date have addressed the optimal extraction methodology specifically for low-microbial abundance clinical samples. Furthermore, due to cost and efficacy, existing comparisons are predominantly based on sequencing data of 16S rDNA conserved regions of bacterial nucleic acids (Bik et al., 2010). It is unclear how extraction methods affect observed microbial community structure and downstream sequencing analyses, particularly with the advent of whole genome sequencing (WGS) and metagenomic analyses.

This paper examines two microbial DNA isolation protocols for human gastric, esophageal, and colorectal biopsies: a mechanical disruption homogenization protocol and a prolonged enzymatic lysis with lysozyme and proteinase K digestion. We compared these two protocols in terms of microbial yield and capture from low abundance anatomic sites by whole genome analysis. From resulting qPCR and WGS data, we conclude that the extraction methods appear similar when determining overall community structure within low abundance gastric and esophageal biopsy samples. Each method identified similar bacterial populations, for example 26 out of the 36 most prevalent bacterial species identified by WGS could be detected using either method. The minimal differences observed in the other 10 species were not in any generalizable fashion and mostly low abundance. The homogenization method does result in higher bacterial content than enzymatic lysis, resulting in higher bacterial read counts, when undergoing WGS.

## Results

We examined a total of 17 biopsy samples from 12 patients, including three gastric samples, four esophageal samples, five colorectal cancer samples and five matching normal colon tissue samples. DNA extracted from these samples, using either the homogenization method or the lysis method, were then compared using qPCR quantitation. Of these, five samples (e.g., three gastric and two esophageal samples) were further examined by WGS.

### qPCR Quantification of All Samples

**Figure 1:**
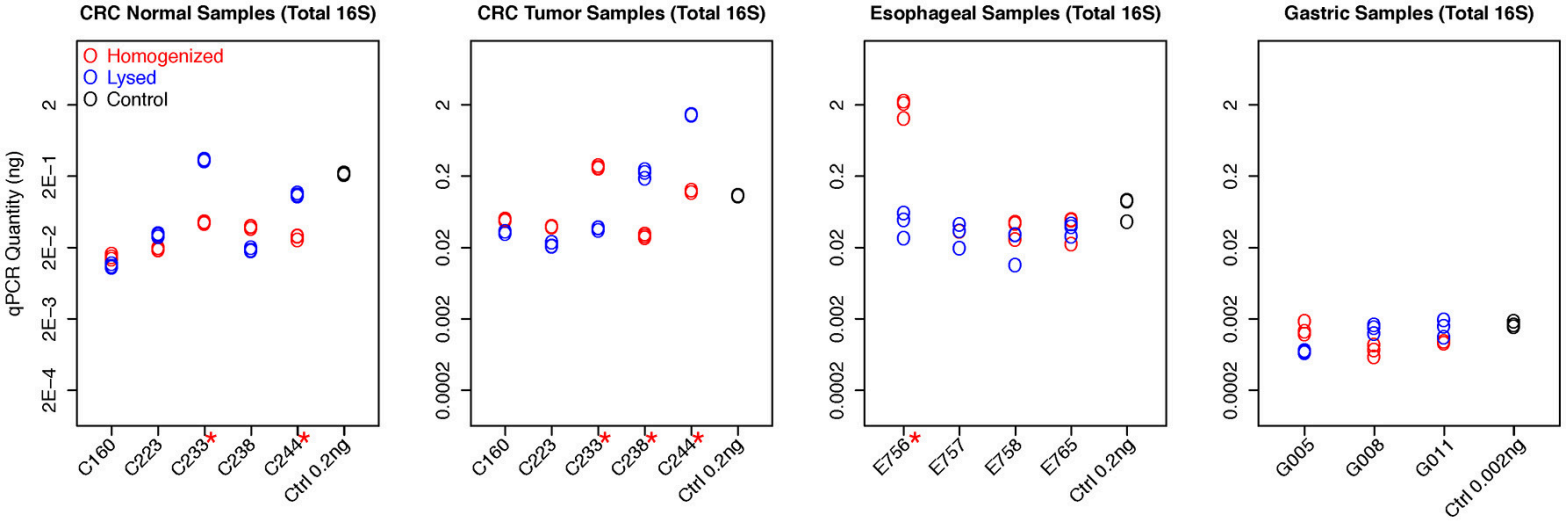
qPCR quantifications of total bacterial content for all samples. The qPCR quantification of each experiment, including replicates, was plotted for each sample with different colors to distinguish two protocols. The readouts of replicates from the same sample are highly consistent. The control samples with known concentration also have been included in all experiments as the reference, demonstrating high accuracy for bacteria quantification. Six samples show significant difference between qPCR quantification of two protocols by student *t*-test (with red * mark).

We first evaluated the total bacterial content from different tissues based on the qPCR quantification measured by universal 16S rDNA primers (Figure 1). Gastric samples had approximately a 10-fold lower bacterial abundance than colorectal and esophageal tissue confirming previous observations (Bik et al., 2006). Total bacterial content from both protocols are highly consistent in most samples. Eleven out of Seventeen samples show no significant difference between qPCR quantification of two protocols by student *t*-test (*p* > 0.05). Although the total bacterial contents are different in six samples (C233-Normal and Tumor, C244-Normal and Tumor, C238-Tumor, and E756) when extracted using homogenization vs. lysis method, no specific extraction protocol provided consistently higher yields. This suggests both methods have comparable efficiency in extracting bacterial DNA from human tissue (Figure 1).

**Figure 2:**
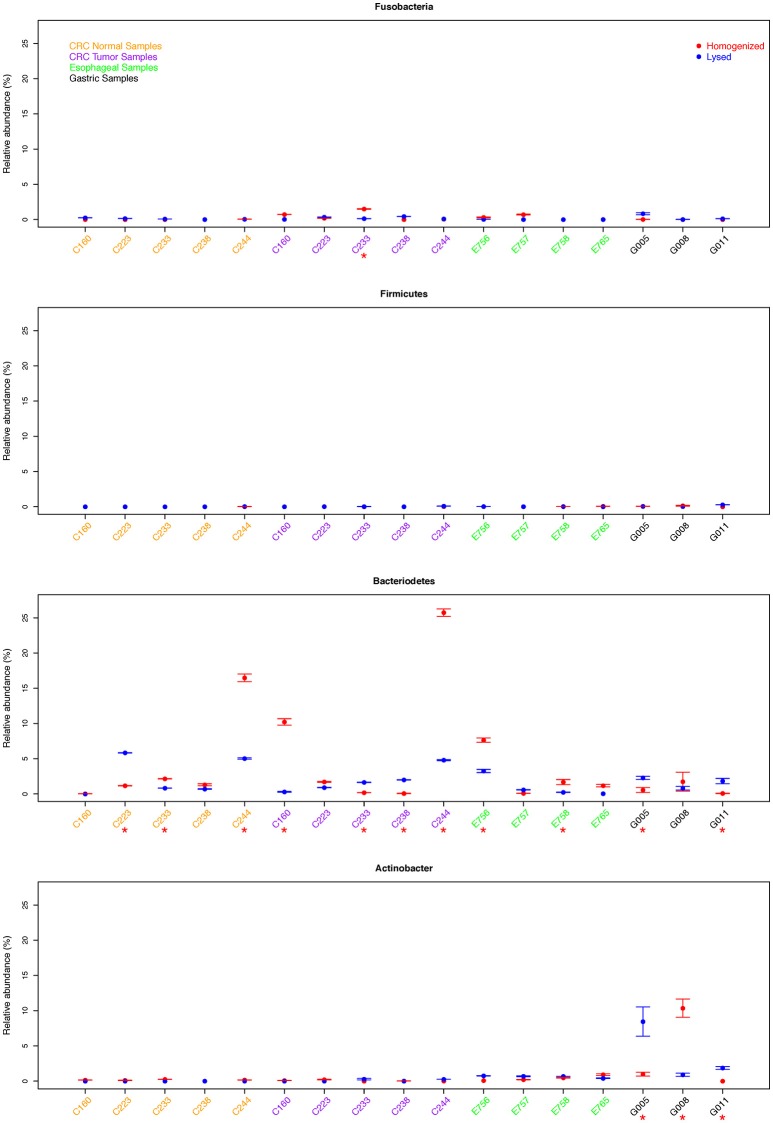
Comparison of relative abundance of *Fusobacterium* sp. and three phyla for all samples. We assessed abundance of each of the above-mentioned bacterial clade relative to total 16S rDNA qPCR quantitation. Samples with significant difference between two protocols were marked with red *.

We then asked if there was a specific preference for either method to extract the Gram-positive or Gram-negative clade. To evaluate this, we used phyla-specific primers to evaluate the extraction of three dominant bacterial phyla in the human GI tract, namely *Actinobacter* (Gram positive), *Firmicutes* (generally Gram positive), and *Bacteroidetes* (Gram negative) using each method (Figure 2). In addition, we also quantified a specific genus *Fusobacterium* which is a common bacterium in GI tract albeit with low relative abundance. We assessed abundance of each of the above-mentioned bacterial clade relative to total 16S rDNA quantitation.

Consistent with low total bacterial content, we found low relative abundance of *Firmicutes* as well as *Fusobacterium* in all gastric cancer samples examined and this difference was not significant between the two extraction methods. Whereas, samples G005 and G011 showed significantly higher abundance of *Bacteroidetes* using the lysis method, the homogenization method yielded a higher *Bacteroidetes* proportion in sample G008. We also found significantly different quantitation of *Actinobacter* in all three gastric samples (*p* < 0.05), although this difference was not consistently in favor of one method or the other (Figure 2). In esophageal samples, we found no significant difference in the relative abundance of *Fusobacterium, Firmicutes*, or *Actinobacter* between the two extraction methods in any of the samples. Only two out of four esophageal samples (E756 and E758) showed a significantly higher abundance of *Bacteroidetes* using the homogenization method which was also in contrast to the lysis method that yielded higher abundance in gastric cancer samples (Figure 2).

The colon tumor and normal samples demonstrated greater variability in bacterial quantification (5 out 10 samples differed significantly, *p* < 0.05 by student *t*-test). Notably, despite these differences in bacterial abundances, there was no trend as to which extraction method would routinely provide the greatest bacterial yield. For example, C233 tumor appears to have significantly greater total bacterial content with the homogenization extraction method (Figure 1), with significantly greater amounts of *Fusobacterium* (9 out of 10 colon samples were not significantly different for *Fusobacterium*) (Figure 2) whereas the lysis method identified higher amounts of *Bacteroidetes*. In contrast, the C233 normal tissue sample seems to have greater bacterial content with the lysis extraction method (Figure 1), whereas significantly more *Bacteroidetes* was identified in this sample using the homogenization method and no difference in quantitation of *Fusobacterium*. Moreover, none of the colon normal and tumor pairs displayed any difference in relative abundance of either *Firmicutes*, or *Actinobacter* (Figure 2). While 7 out the 10 colon samples showed significantly different values for *Bacteroidetes* quantitation, once again, this difference wasn't specific to a particular method of extraction.

Overall, no specific method consistently provides higher yields for either of above phyla/genus, and we found near equal quantitation between two methods of most clades across all three types of cancers. Of the 68 samples tested across four different primer sets, 53 samples show no significant difference in quantitation (*p* > 0.05).

### Comparison of WGS Data and Microbial Identification Between Two Protocols

We next quantified bacterial content by shallow WGS (10X coverage of human genome) of the full DNA content of the biopsy. Read counts and mapping rates to hg19 (95.773% ± 0.309) are comparable among all samples (Table S1). The remaining reads after multiple steps for filtering human DNA are used for microbial identification. The homogenization extraction method yielded a greater proportion of reads that mapped to bacterial species (3 of the 5 samples had a 2-fold or greater proportion of bacterial mapped reads after filtering, Table S1).

**Figure 3:**
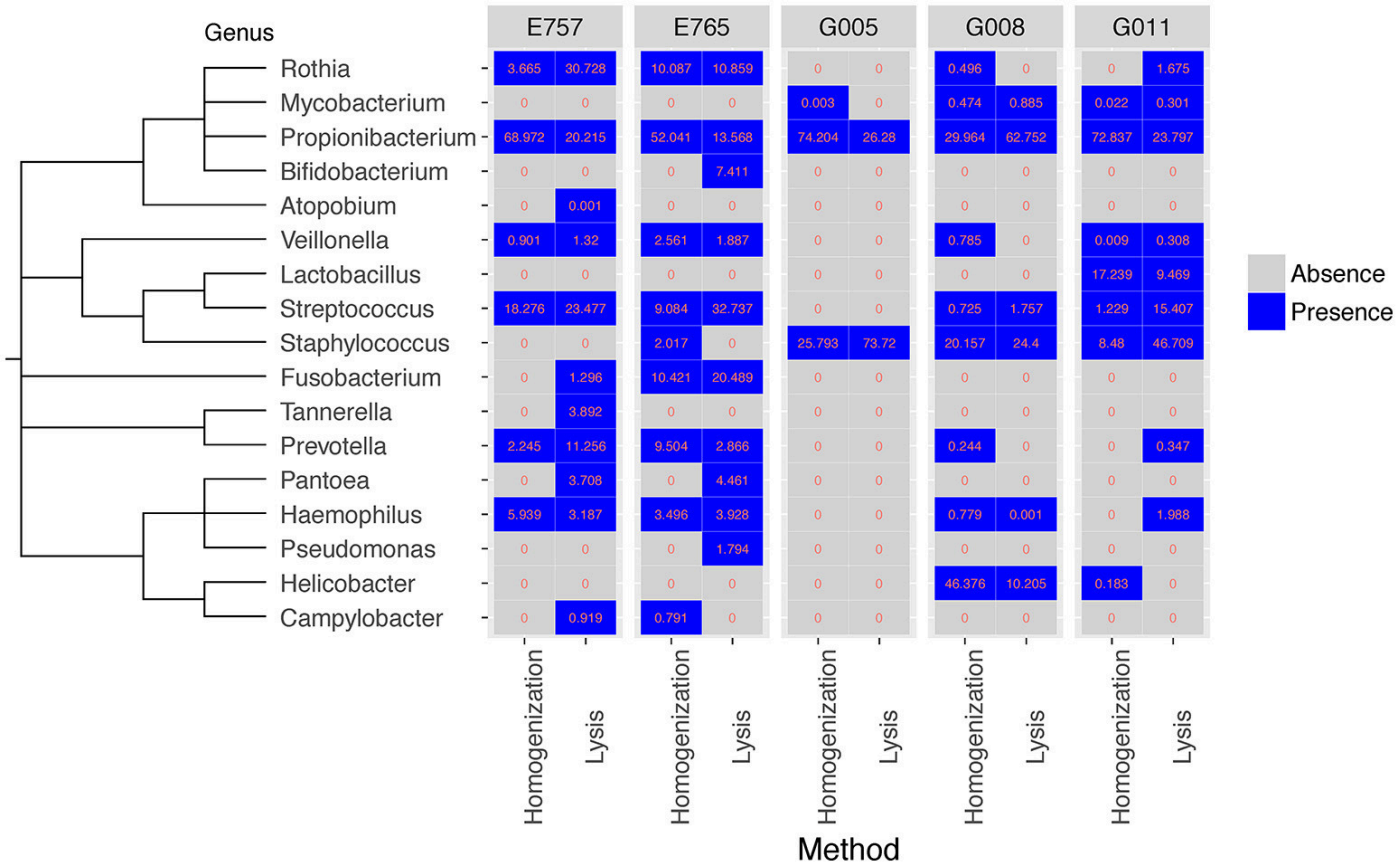
Comparison of bacteria identified from WGS data for five samples at genus level between two extraction methods. Both the homogenization method and the enzymatic lysis method can generate a similar set of microbial genus in all five samples (cosine similarity *p* < 0.05). Values represent percentage of bacteria reads found in each sample.

Overall, both methods were effective at capturing the most prevalent bacterial species within the samples, for example 26 out of the 36 most prevalent bacterial species identified by WGS could be detected using either method (cosine similarity *p* < 0.001) (Table S2). Since the microbial content in biopsy samples is generally low, there may be variable species identification between samples collected from the same patient. We summarized the identification results based on taxonomic genus and order. Considering WGS involves more experimental steps, we expected subtle differences in the community structure from the two methods, and hence we used bacterial absence/presence to measure the profile similarity for the same sample between two different extraction methods. The presence of microbiome at genus level from all five samples, including esophageal tissue E765 and E757, *Helicobacter pylori* negative gastric sample G005, and *H. pylori* positive gastric samples G008 and G011 are all highly consistent between the two extraction methods (cosine similarity *p* < 0.05) (Figure 3). Although, as expected, the precise community structure and abundances between the two methods were different, these differences were specifically limited to the low abundance genus. The homogenizing method was more effective in capturing *H. pylori*, consistent with our previous studies in which the homogenization method can identify the presence of *H. pylori* in gastric mucosa of patients with either active or prior *H. pylori* infection (Zhang et al., 2015). The prolonged lysis method appears to identify *Bifidobacterium* sp. and *Pantoea* sp. to a greater extent (Figure 3).

Overall, Gram-negative and Gram-positive bacteria were represented by both extraction methods with similar efficiency, with some subtle differences (Figure S1). For example, Bacteroidales was detected at low fraction by homogenization method in G008 but not in G011, whereas the detection was inversed in the lysis method. Propionibacteriales is more readily identified using the homogenization method which can yield more reads and higher relative proportion in four out of five samples. Other than Propionibacteriales, there are essentially no other significant difference between any other clades cross all five samples.

## Discussion

Study of the gut microbiome is of increasing importance with numerous studies identifying the role of microbiota in diseases such as cancer. Various protocols have been employed by the scientific community to extract bacterial DNA from human biopsies, which are then analyzed using next generation sequencing methods. In order to ensure that such an analysis of the gut microbiome is not biased due to the method of extraction, we analyzed and compared two widely used DNA extraction protocols, namely the mechanical disruption (homogenization) vs. the enzymatic lysis method. Furthermore, to account for the vast diversity of the human microbiome, it is important that such an analysis is performed on human biopsies, as opposed to preclinical samples. In order to realistically gauge the influence of a particular extraction method on the complexity of the human gut microbiome, we assessed 5 normal and 12 tumor samples across three different cancer types including gastric, esophageal, and colon cancer. Based on qPCR quantification, no single method consistently generated higher total bacterial content. There were a total of six samples that showed significant differences in the overall bacterial content between the homogenization and the lysis method, as assessed by absolute abundance of 16S rDNA using qPCR quantitation. However, this difference was not in favor of one particular extraction protocol. Moreover, it is possible that these differences may be intrinsic, since different regions of the same biopsy were used to extract DNA using the two protocols. To account for these intrinsic differences, we assessed abundance of three different phyla (*Actinobacter, Firmicutes*, and *Bacteroidetes*) as well as *Fusobacterium* relative to total 16S rDNA quantitation. Based on our qPCR results, no single method consistently generated higher readouts for any single clade across all samples. Our results therefore indicate that either extraction method, homogenization or prolonged lysis, can be successfully used to detect bacteria without introducing a significant bias when examining bacteria by qPCR.

We also performed WGS on these samples in parallel to better understand the impact of the extraction method on the bacteria community structure. Whole genome data from gastric and esophageal biopsies did not reveal a significant difference in overall community structure between extraction methods. This is in concordance with studies examining salivary samples and insect gut microbiota whereby 16S rRNA gene amplicons are affected by extraction method but overall community structure is not (Lazarevic et al., 2013; Rubin et al., 2014). In our previous studies, we successfully employed the homogenization method to assess the microbiome composition in gastric mucosa of patients with no infection vs. prior or active *H. pylori* infection (Zhang et al., 2015). Our current analysis further validates that the results obtained previously accurately reflect the predominant bacteria absence/presence in the gastric mucosa, and was not skewed due to the choice of extraction method. However, the relative abundances of some clades do significantly differ by two different methods (Figure S1) as we expected. Considering both read counts and relative proportions, the homogenization extraction method yielded notable higher quantity of Campylobacterales for only sample G008 and Propionibacteriales for four out of five samples. Unlike qPCR or 16S rDNA sequencing which are only quantifying bacteria directly, WGS of biopsies contains extremely high abundance of host DNA which makes bacterial identification challenging (Zhang et al., 2015) and the results very sensitive. Besides two different DNA extraction methods, many other potential confounding factors could easily alter the bacterial relative compositions of WGS, for example, different sequencing batches, different commercial kits, and the microbiome from two split parts of biopsy might not be equally distributed. Then figuring out the exact reasons leading to the bacterial quantification differences between two extraction methods will be very difficult, but even with those variances all predominant clades can be detected by both methods and the quantification of most clades are not in favor of one particular method.

Gram-positive bacteria contain a single layer of peptidoglycan in their cell wall whereas gram-negative organisms contain an additional layer of lipopolysaccharide referred to as the outer membrane. In order to determine, if gram-negative vs. positive bacteria show differential susceptibilities toward lysis or homogenization methods, owing to their different cell wall composition, we compared bacterial yields using qPCR quantitation of 16S rDNA from *Actinobacter* phyla (gram positive), *Firmicutes* (generally gram-positive organisms) and *Bacteroidetes* phyla (gram-negative organisms) relative to total 16S rDNA quantiation. We found very similar yields from *Actinobacter* as well as from other phyla, irrespective of differences in their cell wall composition. Together, our data suggest that both extraction methods can be successfully employed in assessing the overall microbiome composition and content in human biopsies without skewing results due to the choice of extraction method.

The homogenization extraction method does have some favorable characteristics. As a standard protocol for human DNA processing, the homogenizing method has been widely used in many large-scale studies, including large scale cancer project (https://cancergenome.nih.gov/). In our previous study, we successfully retrieved the microbiome information from TCGA samples with our computational pipeline (Zhang et al., 2015). Although the prolonged lysis method might be the preferred way to extract DNA in many microbiome projects (Mann et al., 2014), in many clinical studies, small tissue biopsy samples may not yield sufficient DNA for multiple experiments. For example, separate tissues may be required for both metagenomics analysis with the lysis method and host genomics analysis with the homogenization method. In this study, we show that DNA extraction by the homogenization method is comparable for microbiome profiling as the prolonged enzymatic lysis method. Thus, the homogenization method could allow us to assess microbial and host genomics simultaneously from small clinical biopsy specimens. In addition, the homogenizing method is a faster and more cost-effective method than enzymatic lysis as well as yields higher read counts in WGS. It is further possible that other existing methods such as using a combination of enzymes (lyticase, mutanolysin, lysostaphin) for enzymatic lysis with bead beating process may increase the overall yield and further diversify the community structure (Yuan et al., 2012; Goldschmidt et al., 2014). However, our results suggest that the most abundant bacterial species could still be identified using the above two methods, even in low-bacteria tissue samples. Overall, based on generally equivalent microbiome profiles, but greater versatility for host genomic studies, the homogenizing method on small clinical biopsies or samples with low-bacterial contents for DNA extraction is likely to provide greater tissue utility.

## Materials and Methods

### Sample Collection

This study was performed under one of two clinical tissue acquisition studies approved by the Weill Cornell Medical College Institutional Review Board (IRB). All participants provided written informed consent for use of their tissue samples in accordance with the declaration of Helsinki prior to study enrollment. Patients were enrolled in one of two studies—Weill Cornell Medical College Gastric Cancer and H. pylori Research Database and Tissue Repository (IRB 1203012274), and the NYPH-Weill Cornell Digestive Disease Registry (IRB 0908010582).

#### Colorectal Cancer Samples

A total of five colorectal tumor and matching normal pairs were obtained from the colorectal cancer biobank supported by the Translational Research Program at WCMC Pathology and Laboratory Medicine. For each sample, 3–4 frozen cores of 1.5 mm diameter each were obtained. One core for each of the samples was used for DNA extraction by either the homogenization or the lysis method. The DNA was subsequently used for qPCR and WGS studies.

#### Gastric Samples

Three gastric mucosal biopsy pairs were obtained from the Weill Cornell Medicine Gastric Cancer Center and *H. pylori* Research Database, a registry and tissue repository to examine the natural history of *H. pylori* infection in patients with and without gastric cancer. Gastric biopsy samples were sourced from fundus or proximal body sections of the stomach from patients without gastric carcinoma. Biopsies were obtained using the Bard Precisor EXL® coated disposable biopsy forceps (Bard International, Murray Hill, NJ, USA) and were immediately placed into individual sterile cryovials on dry ice and flash frozen while still in the endoscopic suite. The samples were then transferred to liquid nitrogen for prolonged storage.

#### Esophageal Samples

Esophageal cancer and pre-cancer tissue biopsies were sent to us by our collaborators at University of Kansas School of Medicine, Kansas City, KS. These biopsy samples were obtained from the esophagus at about 2–3 cm above gastroesophageal junction. Tissues were first stored in RNA later solution overnight at 4°C prior to freezing and were shipped to us on dry ice. The DNA from the biopsies was then extracted either using the homogenization method or the enzymatic lysis method as described.

### DNA Extraction

Non-paired biopsies from the same tissue were equally cut prior to gDNA isolation to enable extraction using the two extraction methods illustrated below.

#### Extraction Method 1: Homogenization (Mechanical Disruption)

Frozen biopsy samples (~3–5 mg) were placed in a 2 ml sterile Eppendorf tube containing 350 μl of RLT lysis buffer (Qiagen®). Samples were then homogenized individually on ice for 20–30 s using a pre-sterilized homogenizer (Pro250® Pro Scientific) until tissue was uniformly disrupted. gDNA was subsequently isolated according to manufacturer's instructions from Qiagen Allprep Micro DNA/RNA kit (Qiagen-Hilden, Germany). Briefly, the homogenized lysate were loaded on Allprep DNA mini spin column (Qiagen®) and was centrifuged briefly at 10,000 x g in a table top centrifuge. This step allows binding of DNA to the column. After subsequent washing steps with buffers AW1 (Qiagen®) and AW2 (Qiagen®), DNA is eluted using the elution buffer provided in the kit. Homogenizer was cleaned and sterilized before and after each individual sample with separate 15 ml falcon tubes containing 10% bleach, 70% EtOH, and RNAse/DNAse free sterile water.

#### Extraction Method 2: Enzymatic Lysis and Incubation

Frozen biopsy tissue (~3–5 mg) was placed in a 1.5 ml centrifuge tube and 180 μl of lysozyme (Gold Biotechnology Cat no: L-040-10) (20 mg/ml stock concentration prepared in TE) was added. Lysozyme was pre-warmed to 37°C prior to its addition to the sample. Samples were then incubated at 37°C for 1 h. Proteinase K (20 μl of 20 mg/ml stock concentration; Thermo Scientific Cat no: EO0491) was added after the incubation. And sample was incubated at 56°C on a heat block and periodically vortexed until complete lysis of tissue was observed (4–6 h). Samples were then incubated in 200 μl Buffer AL from QIAamp DNA Mini kit (Qiagen) for 30 min at 70°C before continuing extraction using QIAamp DNA mini kit (Qiagen) according to manufacturer's instructions (Viljoen et al., 2015). Briefly, after the 30 min incubation at 70°C, sample was loaded on QIAamp DNA spin column (Qiagen®) and was centrifuged briefly at 10,000 x g in a table top centrifuge. The column is then washed with buffers AW1 (Qiagen®) and AW2 (Qiagen®) and DNA is eluted using the elution buffer provided in the kit.

### Quantitation and Quality Control

DNA concentrations and quality were measured using the Qubit™ dsDNA high sensitivity assay kit (Invitrogen®) and a Qubit™ 2.0 Fluorometer (Life Technologies, Grand Island, NY, USA).

To validate that the two kits used in the above two mentioned protocols themselves did not contribute to any variation, we re-extracted 1 μg of DNA from one of the gastric cancer samples using Allprep DNA/RNA micro kit as well as QIAamp DNA mini kit. This re-purified DNA from the two kits (5 ng each) was then subjected to qPCR using the 16S rDNA gene primer set to check for bacterial abundance. Furthermore, to prove that the columns itself were not a source of any contamination, we performed parallel purifications using water and subjected the eluate to qPCR against the 16S rDNA gene primer set (Figure S2).

### Real-time PCR (qPCR)

To corroborate total bacterial abundance (measured by highly conserved 16S rDNA sequences) and *Fusobacterium* sp. abundances, genomic DNA was initially analyzed via qPCR. Reactions were carried out on a StepOne® 48-well thermocycler (Applied Biosystems). Primers used were FAM-labeled Genesig® 16S Eubacteria quantification primer/probe set (Proprietary sequence-PrimerDesign®) to determine total bacterial composition and a custom FAM-MGB *Fusobacterium* primer/probe (Applied biosystems) for *Fusobacterium* quantitation: Forward: 5′–AAGCGCGTCTAGGTGGTTATGT-3′; Reverse: 5′-TGTAGTTCCGCTTACCTCTCCAG-3′; Probe:FAM CAACGCAATACAGAGTTGAGCCCTGCATT (Martin et al., 2002). Phylla-specific primers used for detection of organisms from *Bacteroidetes* were adapted from (Yang et al., 2015) and those for *Actinobacter* and *Firmicutes* were adapted from (Pfeiffer et al., 2014). All three of these primer sets have been validated for both specificity and sensitivity toward each of these phylla in these above-mentioned manuscripts.

Real-time PCR reactions were performed in triplicate on MicroAmp® fast-optical 48 well (0.1 ml) reaction plates (Applied Biosystems). Each reaction contained 1–2 μl template DNA, 0.5ul of 20X ABI primer/probe assay, 5 μl Taqman® Gene Expression Mastermix (Applied Biosystems), and RNase/DNase free water to a total volume of 10 μl

Cycling conditions included an initial denaturation at 50°C for 2 min and 95°C for 10 min followed by 40 cycles of 95°C for 15 s and 60°C for 1 min.

Absolute quantification was performed using a standard reference curve and controls prepared using known concentrations *Escherichia coli* gDNA (ATCC®) for 16S total quantitation and *Fusobacterium nucleatum* gDNA (ATCC® Strain VPI-4355) for *Fusobacterium* sp. quantitation.

### Whole Genome Sequencing (WGS)

Genomic DNA (200–500 ng) was submitted from each sample to the Weill Cornell Medicine Epigenomics Core for library preparation and subsequent WGS using an Illumina TruSeq DNA-seq® DNA sample preparation kit and the Illumina HiSeq 2500® platform. Each sample was sequenced on a single flow cell lane as 50-bp paired-end reads at roughly 10x coverage to Human genome. Homopolymers, adapters and distribution of base quality of raw sequences from each sample were investigated using FastQC (version 0.10.1). In order to estimate the potential contaminations from experiment, two control samples containing only RNase/DNase free water went through two extraction procedures along with biopsies samples, and were similarly sequenced as the clinical samples. There were no bacteria detected by either qPCR or WGS in those two control samples.

### Computational Pipeline for Bacterial Identification

The bacterial content of each WGS sample was identified with our in-house computational pipeline (Zhang et al., 2015), which is designed to quantify microbiome from WGS data of small clinical sample with following steps:

1) Filtering human DNA: All reads from WGS data were aligned to corresponding Human DNA databases with four different aligners (BWA, RepeatMasker, BLAST, MegaBlast) step by step. Any reads mapped to Human DNA databases were removed and the remaining reads were used as the input for bacteria identification.
2) Mapping to bacterial genomes: 1,421 non-redundant bacterial genomes were collected from NCBI. Bowtie2 was used as the aligner to map the read to each bacterial genome, and each read was labeled as either uniquely mapped, unmapped or ambiguous (multiple genomic mapping).
3) Genome coverage evaluation: For any bacteria with more than ten uniquely mapped reads we compute a genomic coverage measure to further remove false identifications.
4) Calculating the relative abundance: Based on the identified bacteria from step 3, we applied a Bayesian statistical framework to assign all mapped reads to the most probable bacterial source genome (Wood and Salzberg, 2014). Finally, the relative abundances of bacteria were calculated for each sample.

### Statistical Analyses

In qPCR data analysis, to determine the total bacterial content difference in particular sample between two extraction methods, we calculated absolute[log2(Homogenization qPCR readout/Lysis qPCR readout)] for each experiment and each sample individually, and then performed a one-tailed one-sample *t*-test with H0: μ < 2 across all samples. Statistical significance was defined as *p* ≤ 0.05. For each particular phyla/genus, we assessed relative abundance difference between two extraction methods, and then performed a one-tailed one-sample *t*-test with H0: μ < 1% across all samples. Statistical significance was defined as *p* ≤ 0.05. For WGS data, cosine similarity was applied to measure the similarity of microbiome absence/presence detection between two extraction methods, and corresponding permutation test was used to evaluate statistical significance (Smirnov et al., 2016).

## Author Contributions

MS, DB, and CZ conceived and designed project. PT and SP extracted DNA and performed the experiments. PS and SV collected clinical samples. CZ and DB performed the bioinformatic analyses. MS, PT, CZ, and DB interpreted the results. CZ, PT, SP, DB, and MS wrote the manuscript. All authors contributed to final revisions of the manuscript.

### Conflict of Interest Statement

The authors declare that the research was conducted in the absence of any commercial or financial relationships that could be construed as a potential conflict of interest.

## Supplementary Material

Figure S1 Comparison of bacteria identified from WGS data for five samples at order level between two extraction methods (Similar to Figure 3).

Figure S2 Comparison of the Qiagen Allprep Micro DNA/RNA kit used with the homogenization protocol and the QIAamp DNA mini kit (Qiagen) that was employed with the Lysis protocol. Overall bacterial abundance (ng) using 16S rDNA primer set is represented. The two different kits used with two different extraction protocols themselves did not contribute to any differences as demonstrated by equal abundance of bacterial DNA in the sample re-extracted using the two kits.

Table S1 Mapping statistics of WGS samples.

Table S2 Species-level identification results of WGS samples.

## References

[B1] Bik E. M. Eckburg P. B. Gill S. R. Nelson K. E. Purdom E. A. Francois F.. (2006). Molecular analysis of the bacterial microbiota in the human stomach. Proc. Natl. Acad. Sci. U.S.A. 103, 732–737. 10.1073/pnas.0506655103 16407106 PMC1334644

[B2] Bik E. M. Long C. D. Armitage G. C. Loomer P. Emerson J. Mongodin E. F.. (2010). Bacterial diversity in the oral cavity of 10 healthy individuals. ISME J. 4, 962–974. 10.1038/ismej.2010.30 20336157 PMC2941673

[B3] Crandall K. A. Freishtat R. J. Pérez-Losada M. (2016). Comparison of two commercial DNA extraction kits for the analysis of nasopharyngeal bacterial communities. AIMS Microbiol. 2, 108–119. 10.3934/microbiol.2016.2.108

[B4] Goldschmidt P. Degorge S. Merabet L. Chaumeil C. (2014). Enzymatic treatment of specimens before DNA extraction directly influences molecular detection of infectious agents. PLoS ONE 9:e94886. 10.1371/journal.pone.0094886 24936792 PMC4061000

[B5] Human Microbiome Project C. (2012). Structure, function and diversity of the healthy human microbiome. Nature 486, 207–214. 10.1038/nature11234 22699609 PMC3564958

[B6] Lazarevic V. Gaia N. Girard M. Francois P. Schrenzel J. (2013). Comparison of DNA extraction methods in analysis of salivary bacterial communities. PLoS ONE 8:e67699. 10.1371/journal.pone.0067699 23844068 PMC3701005

[B7] Mann E. Pommer K. Mester P. Wagner M. Rossmanith P. (2014). Quantification of Gram-positive bacteria: adaptation and evaluation of a preparation strategy using high amounts of clinical tissue. BMC Vet Res. 10:53. 10.1186/1746-6148-10-53 24589061 PMC4015715

[B8] Martin F. E. Nadkarni M. A. Jacques N. A. Hunter N. (2002). Quantitative microbiological study of human carious dentine by culture and real-time PCR: association of anaerobes with histopathological changes in chronic pulpitis. J. Clin. Microbiol. 40, 1698–1704. 10.1128/JCM.40.5.1698-1704.2002 11980945 PMC130955

[B9] Moore E. Arnscheidt A. Krüger A. Strömpl C. Mau M. (2004). Section 1 update: Simplified protocols for the preparation of genomic DNA from bacterial cultures, in Molecular Microbial Ecology Manual, eds Kowalchuk G. A. De Bruijn F. J. Head I. M. Akkermans A. D. Van Elsas J. D. (Dordrecht: Springer Netherlands), 1905–1919.

[B10] Pfeiffer S. Pastar M. Mitter B. Lippert K. Hackl E. Lojan P.. (2014). Improved group-specific primers based on the full SILVA 16S rRNA gene reference database. Environ. Microbiol. 16, 2389–2407. 10.1111/1462-2920.12350 25229098

[B11] Rossmanith P. Wagner M. (2011). Aspects of systems theory in the analysis and validation of innovative molecular-biological based food pathogen detection methods. Trends Food Sci. Technol. 22, 61–71. 10.1016/j.tifs.2010.12.004

[B12] Rubin B. E. Sanders J. G. Hampton-Marcell J. Owens S. M. Gilbert J. A. Moreau C. S. (2014). DNA extraction protocols cause differences in 16S rRNA amplicon sequencing efficiency but not in community profile composition or structure. Microbiologyopen 3, 910–921. 10.1002/mbo3.216 25257543 PMC4263514

[B13] Shreiner A. B. Kao J. Y. Young V. B. (2015). The gut microbiome in health and in disease. Curr. Opin. Gastroenterol. 31, 69–75. 10.1097/MOG.0000000000000139 25394236 PMC4290017

[B14] Smirnov P. Safikhani Z. El-Hachem N. Wang D. She A. Olsen C.. (2016). PharmacoGx: an R package for analysis of large pharmacogenomic datasets. Bioinformatics 32, 1244–1246. 10.1093/bioinformatics/btv723 26656004

[B15] Viljoen K. S. Dakshinamurthy A. Goldberg P. Blackburn J. M. (2015). Quantitative profiling of colorectal cancer-associated bacteria reveals associations between fusobacterium spp., enterotoxigenic Bacteroides fragilis (ETBF) and clinicopathological features of colorectal cancer. PLoS ONE 10:e0119462. 10.1371/journal.pone.0119462 25751261 PMC4353626

[B16] Wood D. E. Salzberg S. L. (2014). Kraken: ultrafast metagenomic sequence classification using exact alignments. Genome Biol. 15:R46. 10.1186/gb-2014-15-3-r46 24580807 PMC4053813

[B17] Wu G. D. Lewis J. D. Hoffmann C. Chen Y. Y. Knight R. Bittinger K.. (2010). Sampling and pyrosequencing methods for characterizing bacterial communities in the human gut using 16S sequence tags. BMC Microbiol. 10:206. 10.1186/1471-2180-10-206 20673359 PMC2921404

[B18] Yang Y. W. Chen M. K. Yang B. Y. Huang X. J. Zhang X. R. He L. Q.. (2015). Use of 16S rRNA gene-targeted group-specific primers for real-time PCR analysis of predominant bacteria in mouse feces. Appl. Environ. Microbiol. 81, 6749–6756. 10.1128/AEM.01906-15 26187967 PMC4561689

[B19] Yuan S. Cohen D. B. Ravel J. Abdo Z. Forney L. J. (2012). Evaluation of methods for the extraction and purification of DNA from the human microbiome. PLoS ONE 7:e33865. 10.1371/journal.pone.0033865 22457796 PMC3311548

[B20] Zhang C. Cleveland K. Schnoll-Sussman F. Mcclure B. Bigg M. Thakkar P.. (2015). Identification of low abundance microbiome in clinical samples using whole genome sequencing. Genome Biol. 16:265. 10.1186/s13059-015-0821-z 26614063 PMC4661937

