# Supplementary figures and images for: A Comparison of Homogenization vs. Enzymatic Lysis for Microbiome Profiling in Clinical Endoscopic Biopsy Tissue Samples
Source: Front Microbiol. 2019 Jan 8;9:3246. doi: 10.3389/fmicb.2018.03246 (PMC6331478; doi:10.3389/fmicb.2018.03246)

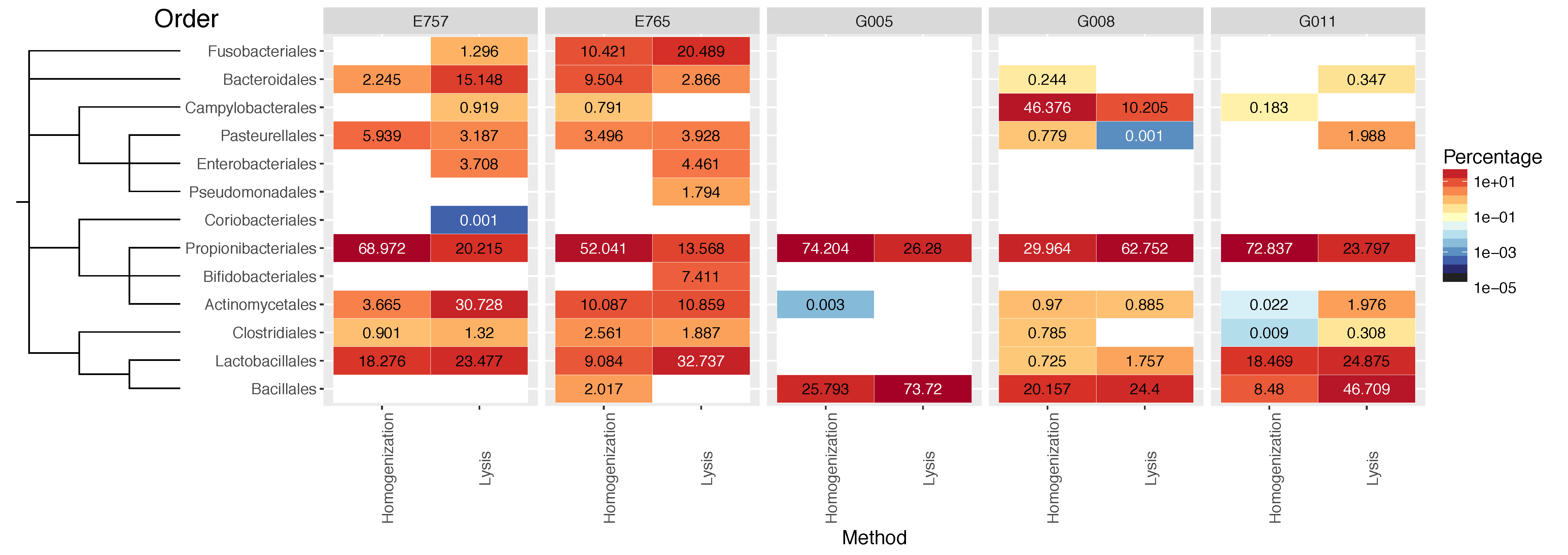

Supplement: Figure S1 — Comparison of bacteria identified from WGS data for five samples at order level between two extraction methods (Similar to Figure 3). [file Image_1.TIFF]

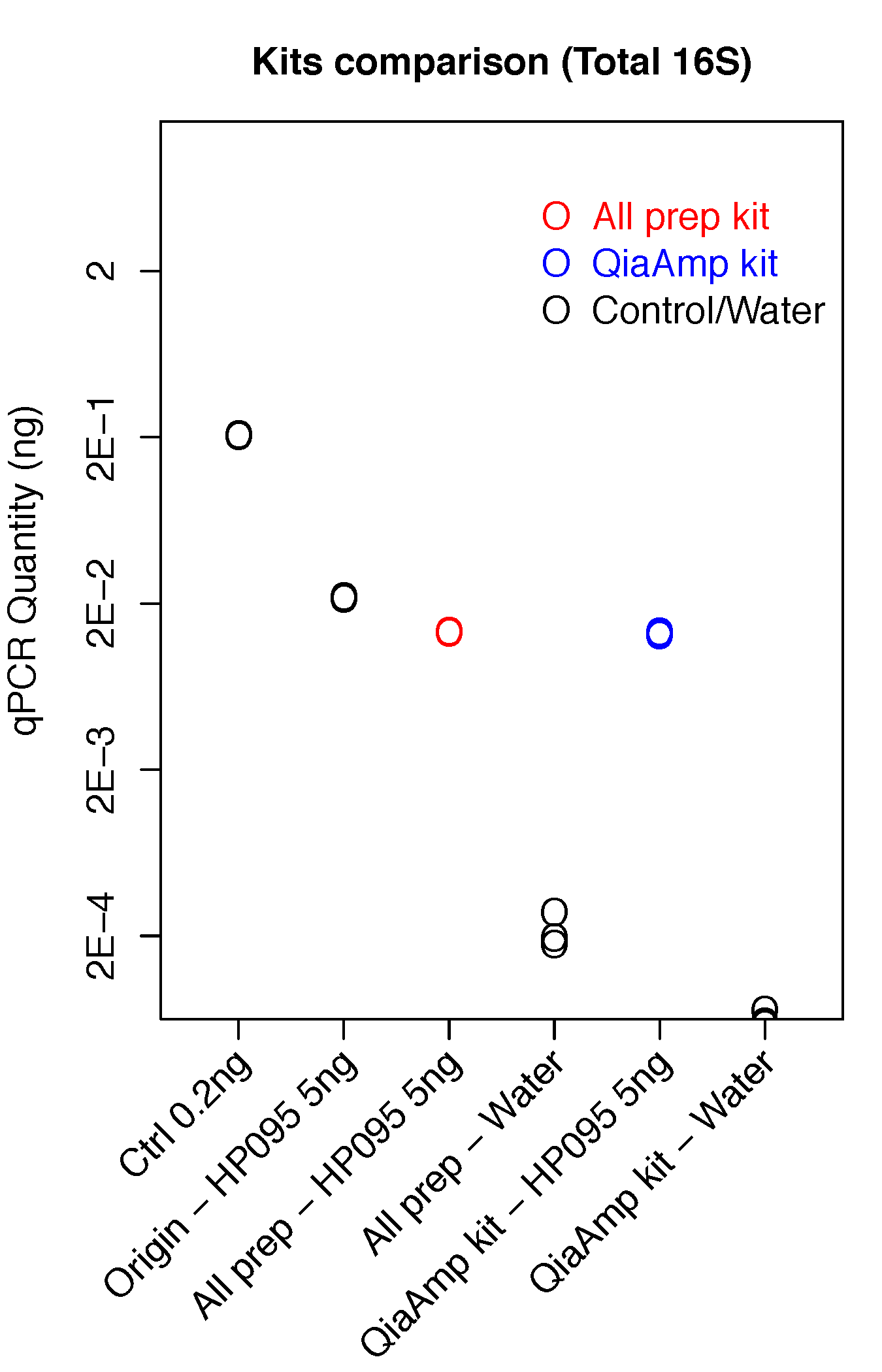

Supplement: Figure S2 — Comparison of the Qiagen Allprep Micro DNA/RNA kit used with the homogenization protocol and the QIAamp DNA mini kit (Qiagen) that was employed with the Lysis protocol. Overall bacterial abundance (ng) using 16S rDNA primer set is represented. The two different kits used with two different extraction protocols themselves did not contribute to any differences as demonstrated by equal abundance of bacterial DNA in the sample re-extracted using the two kits. [file Image_2.TIFF]
